# Supplementary material for: Slideflow: deep learning for digital histopathology with real-time whole-slide visualization
Source: BMC Bioinformatics. 2024 Mar 27;25:134. doi: 10.1186/s12859-024-05758-x (PMC10967068; doi:10.1186/s12859-024-05758-x)
Supplement: Supplementary file 1 — Additional file 1. Supplementary Figures 1–11 and Supplementary Tables 1–3. [file 12859_2024_5758_MOESM1_ESM.docx]

**Supplementary Materials**

**Contents**

**Supplementary Figure 1**

**Supplementary Figure 2**

**Supplementary Figure 3**

**Supplementary Figure 4**

**Supplementary Figure 5**

**Supplementary Figure 6**

**Supplementary Figure 7**

**Supplementary Figure 8**

**Supplementary Figure 9**

**Supplementary Figure 10**

**Supplementary Figure 11**

**Supplementary Table 1**

**Supplementary Table 2**

**Supplementary Table 3**


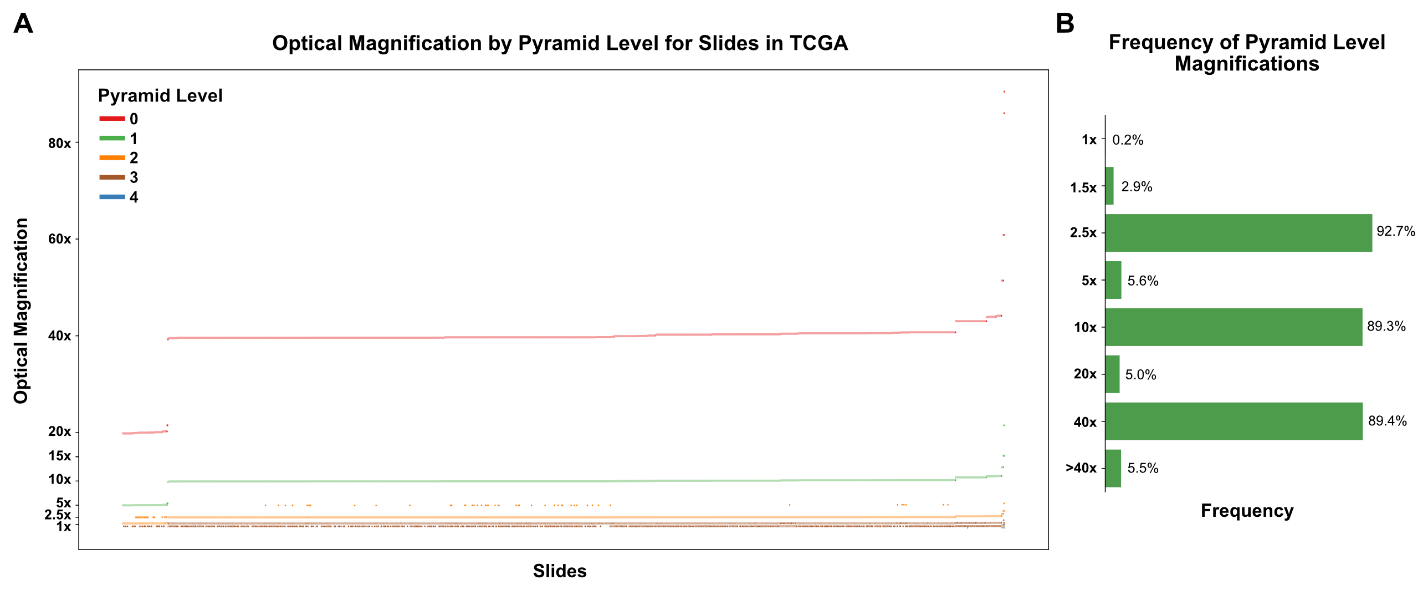


**Supplementary Figure 1. Optical magnification of pyramid layers in slides from The Cancer Genome Atlas.** (a) Effective optical magnification was assessed for pyramid layers in 8,462 slides from The Cancer Genome Atlas (TCGA). Optical magnification was determined from a layer’s micron-per-pixel (MPP) value, using the formula 4 / MPP, where a MPP of 1.0 corresponds to 10x optical magnification and an MPP of 0.25 corresponds to 40x. The majority of slides had an optical magnification of 40x at the highest-resolution pyramid level (level 0), although a subset of slides had a greatest magnification of 20x, and a smaller subset had a magnification exceeding 40x. (b) Frequency of slides with a pyramid layer matching each of the given optical magnifications. The most frequent optical magnification present in a pyramid layer was 2.5x, with 92.7% of slides having a pyramid layer at this magnification.


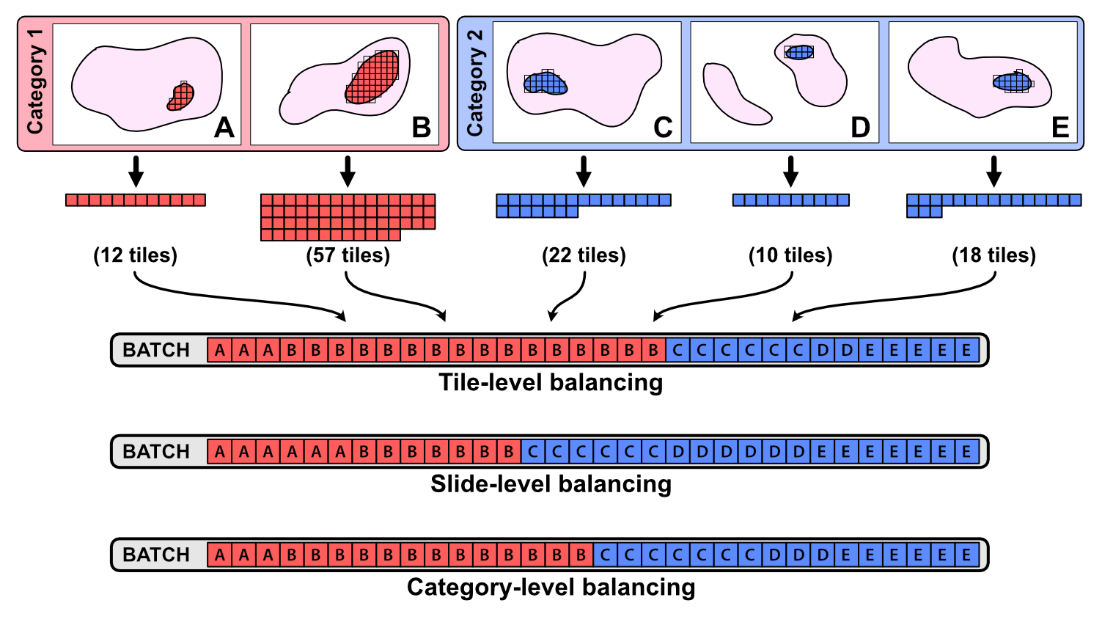


**Supplementary Figure 2. Mini-batch balancing strategies.** When assembling a batch of images for training, images may be sampled in a manner which balances the slides or outcomes represented in a given batch. Tile-level balancing simulates random selection from all tiles in the dataset; slides with more image tiles will be over-represented. Slide-level balancing ensures each slide is equally represented in a training batch, although outcomes with more slides will be over-represented. Category-level balancing results in equal proportions of all outcome categories in each training batch, but may over-represent slides with many tiles and under-represent slides with fewer tiles. The default mini-batch balancing strategy used during training is category-level balancing for when training for a categorical outcome, and slide-level balancing when training a regression model.

**
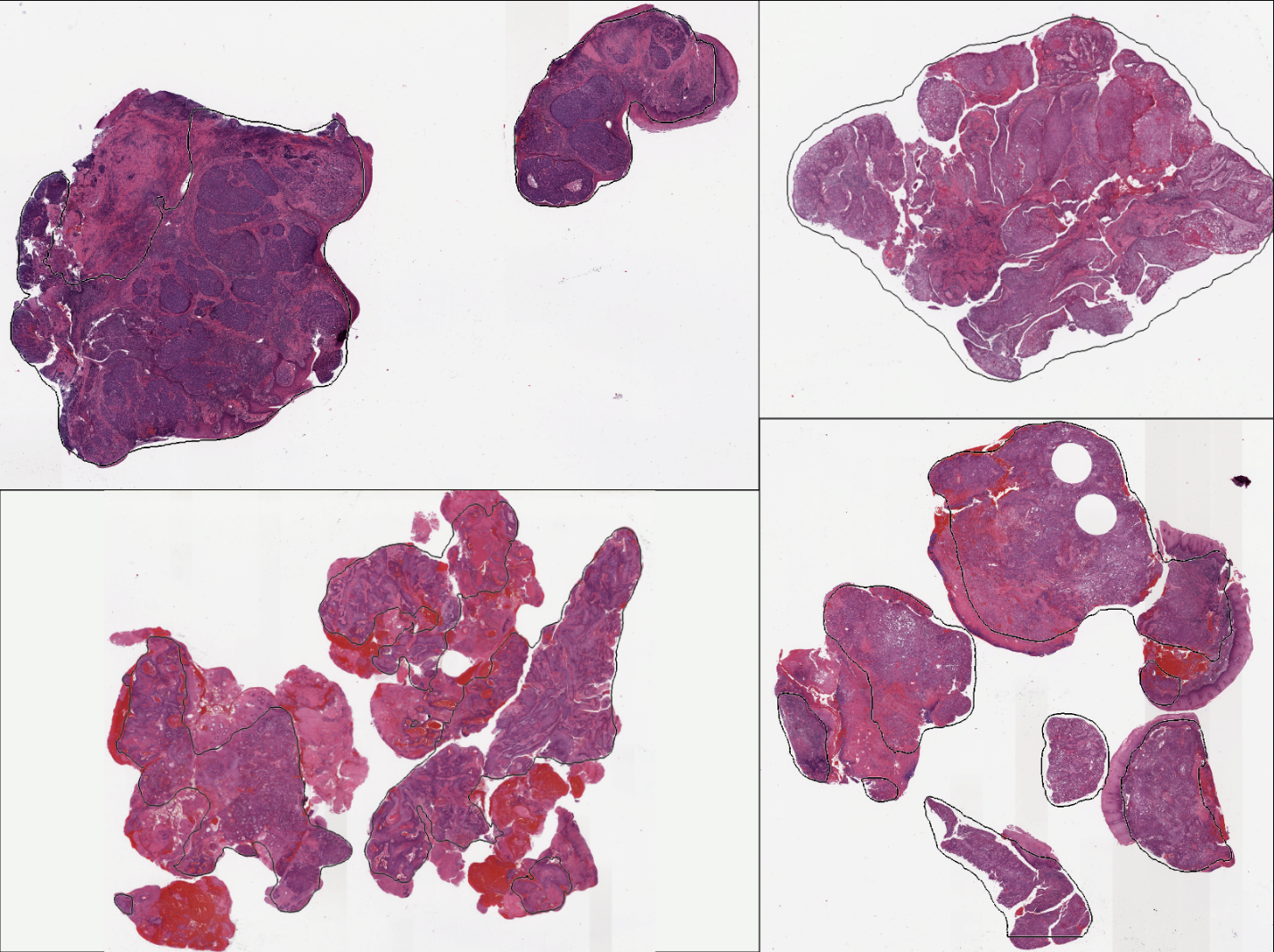
**

**Supplementary Figure 3. Automated tumor identification with a pretrained segmentation model.** A pretrained tumor identification tissue segmentation model, trained on 8,122 slides and paired pathologist-annotated regions of interest (ROIs), has been distributed on Hugging Face. This model was deployed on four whole-slide images of head and neck squamous cell carcinoma from the University of Chicago, with automated tumor ROIs generated as shown above.


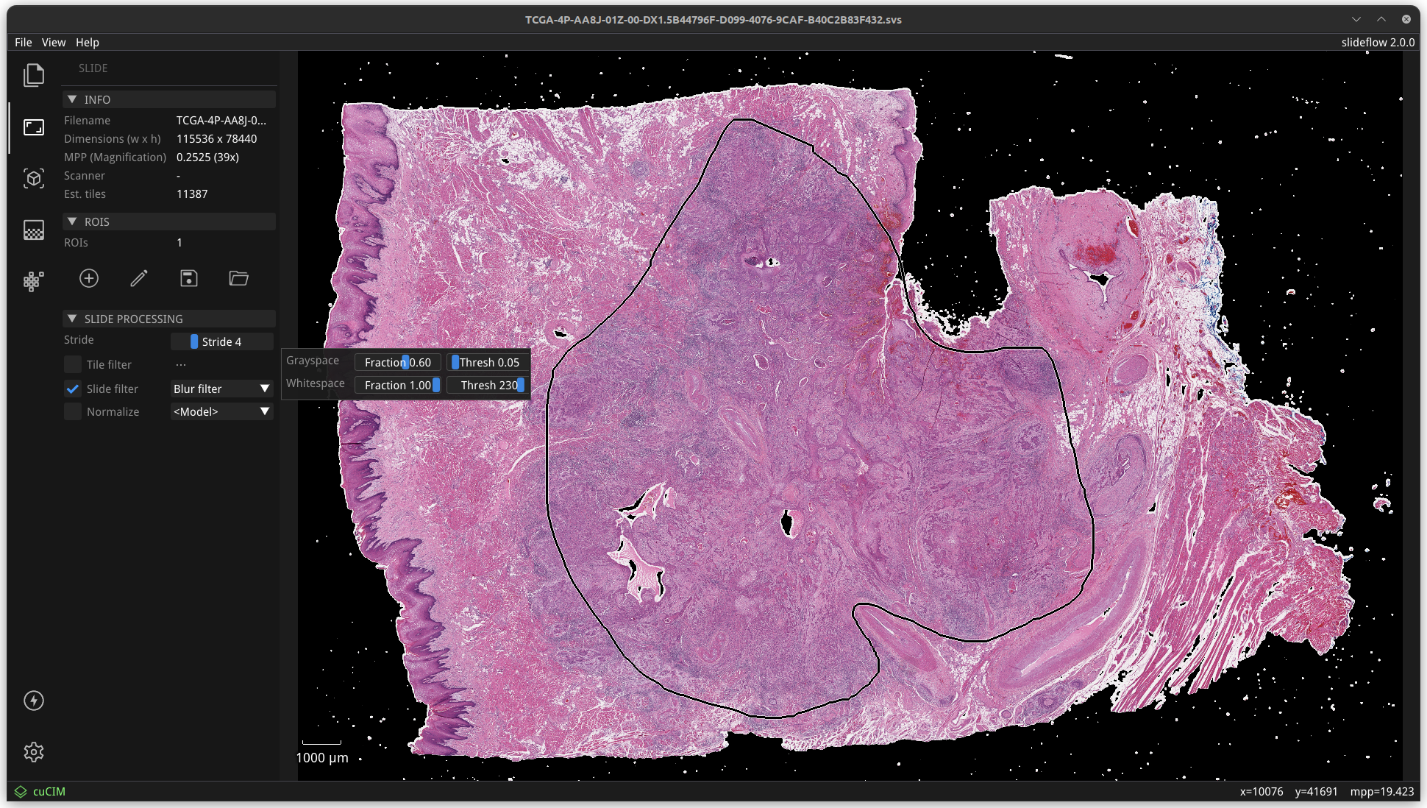


**Supplementary Figure 4. Real-time slide processing preview with Workbench.** Slide processing settings - including Otsu’s thresholding, Gaussian blur filtering, grayspace filtering, and whitespace filtering – can be dynamically previewed in a whole-slide image navigation interface. The control panel on the left is used to select the tile- or slide-level processing parameters. Sliders are used to fine-tune the grayspace and whitespace thresholds. This control panel can also be used to preview and compare stain normalization strategies.


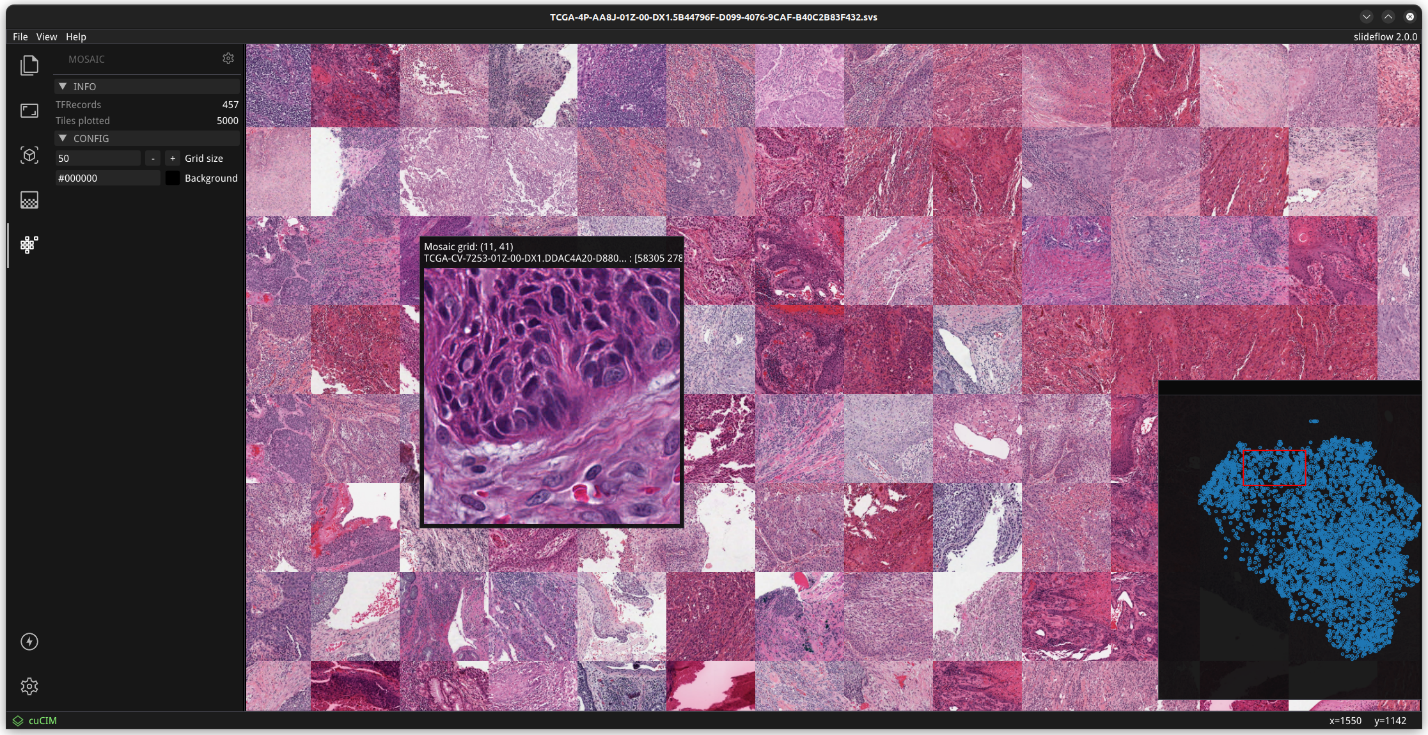


**Supplementary Figure 5. Interactive mosaic map navigation.** Workbench can display exported mosaic maps, supporting panning (with click-and-drag) and zooming (via mouse wheel) for magnified display. Hovering over an image tile will open a preview window showing a larger corresponding area from the whole slide image, with the associated slide name and location coordinates. A UMAP preview is shown in the bottom right corner, indicating the current section of the mosaic map in view. Pressing **Ctrl + =** will increase the size of the mosaic grid by 50%, and pressing **Ctrl + -** will decrease the size of the mosaic grid by 50%.


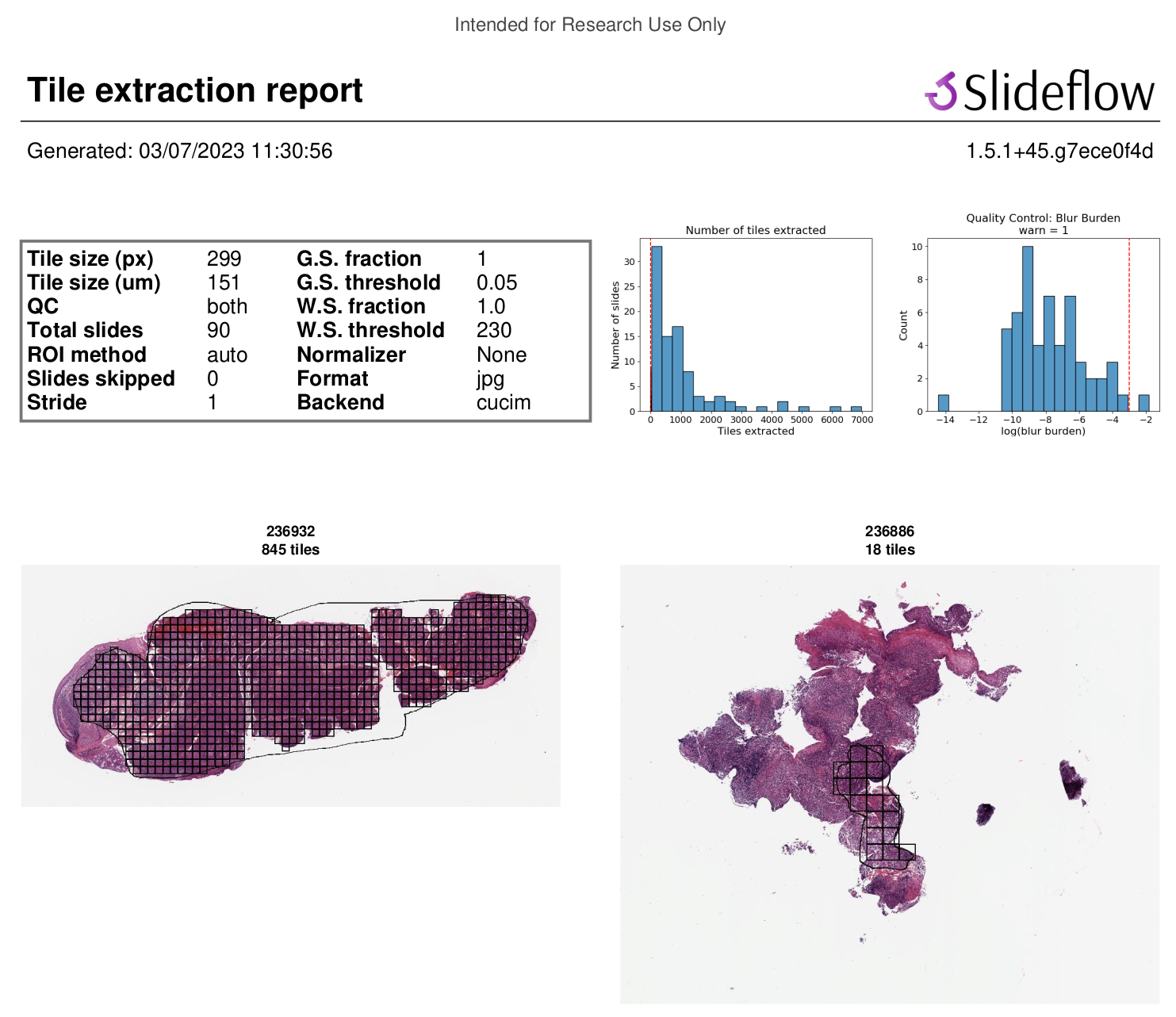


**Supplementary Figure 6. First page from a sample tile extraction PDF report.** After tile extraction, a PDF report is saved summarizing the slide processing parameters, output format, and tiles extracted for each slide. For each slide, a thumbnail of the WSI is shown with Regions of Interest (ROI) (if applicable), and boxes drawn on the thumbnail corresponding to each extracted tile. The total number of tiles extracted for each slide is shown above each thumbnail preview. The first page of the PDF report will include a histogram of the number of tiles extracted for each slide in the dataset.

**
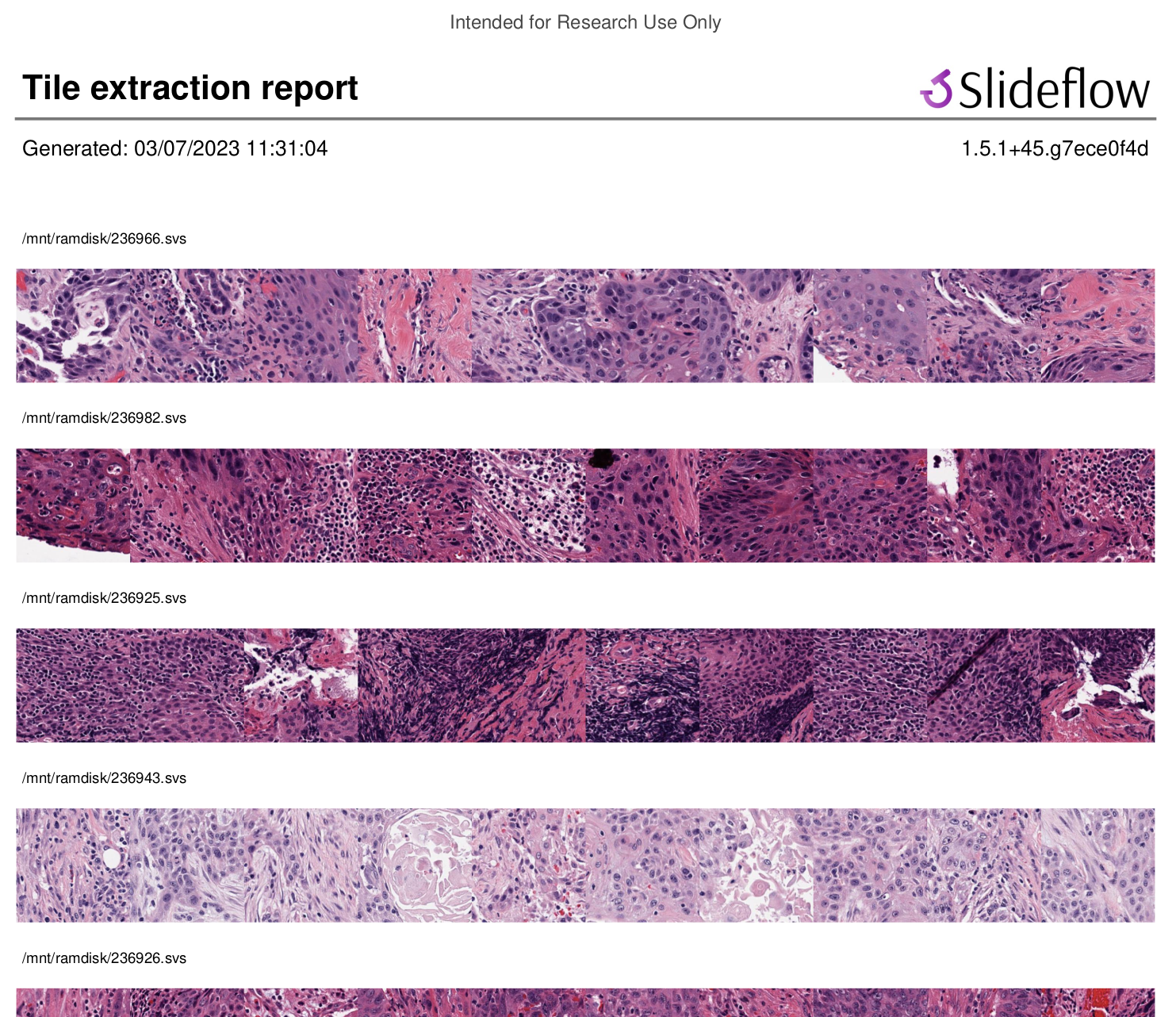
**

**Supplementary Figure 7. Example page from a sample tile extraction PDF report.** The second half of the tile extraction PDF report contains a row for each slide, showing up to 10 image tiles extracted from the slide. This allows the researcher to quickly scan through the dataset of extracted tiles and search for any potential issues, such as highly variable staining patterns; pen marks or artifact; or highly out-of-focus slides.


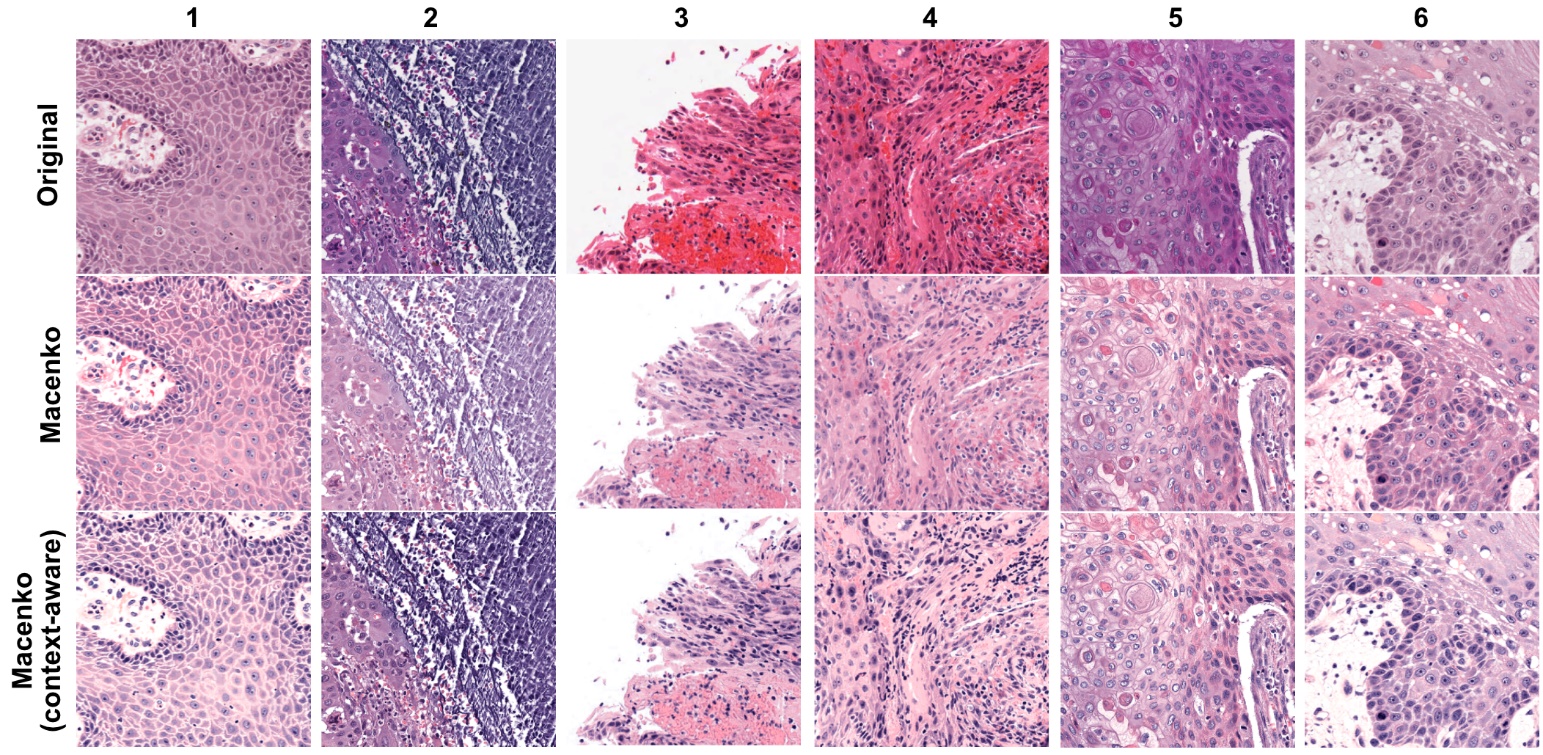


**Supplementary Figure 8. Standard vs. context-aware Macenko normalization.** Six image tiles with different staining patterns are shown before and after stain normalization using standard Macenko and context-aware Macenko stain normalization. In both cases, images are shown using the default “v3” normalization fit.


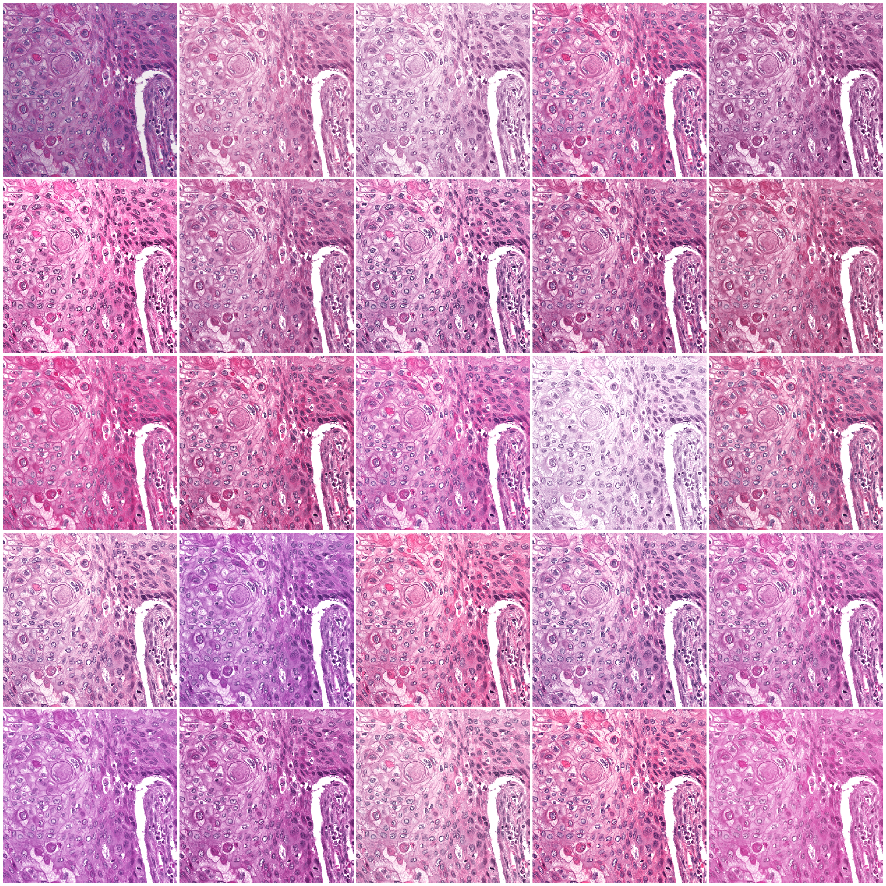


**Supplementary Figure 9. Example images generated using Macenko stain augmentation.** An example original image tile is provided in the top-left corner. The remaining images were generated through a combination of Macenko stain normalization and augmentation, where images are fit to a randomized stain matrix target around the default “v3” normalization fit.

**
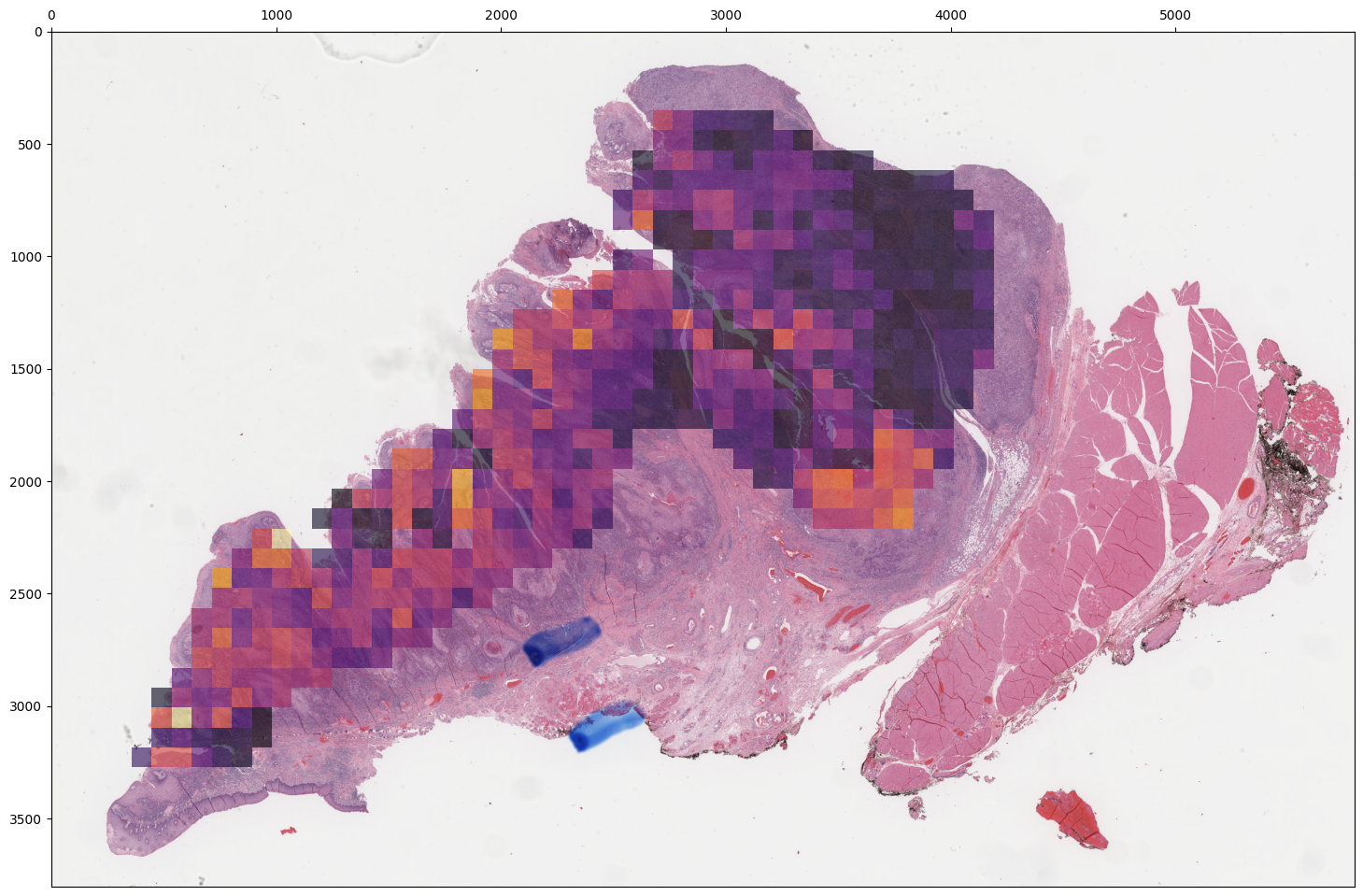
**

**Supplementary Figure 10. Attention heatmap generated from the multiple-instance learning (MIL) model.** An attention heatmap from the final multiple-instance learning model was generated for the same slide as shown in Fig. 13. In general, the areas with strong predictions and high-confidence from the tile-based model, as shown in Fig. 13, also have high attention in this MIL heatmap.

**
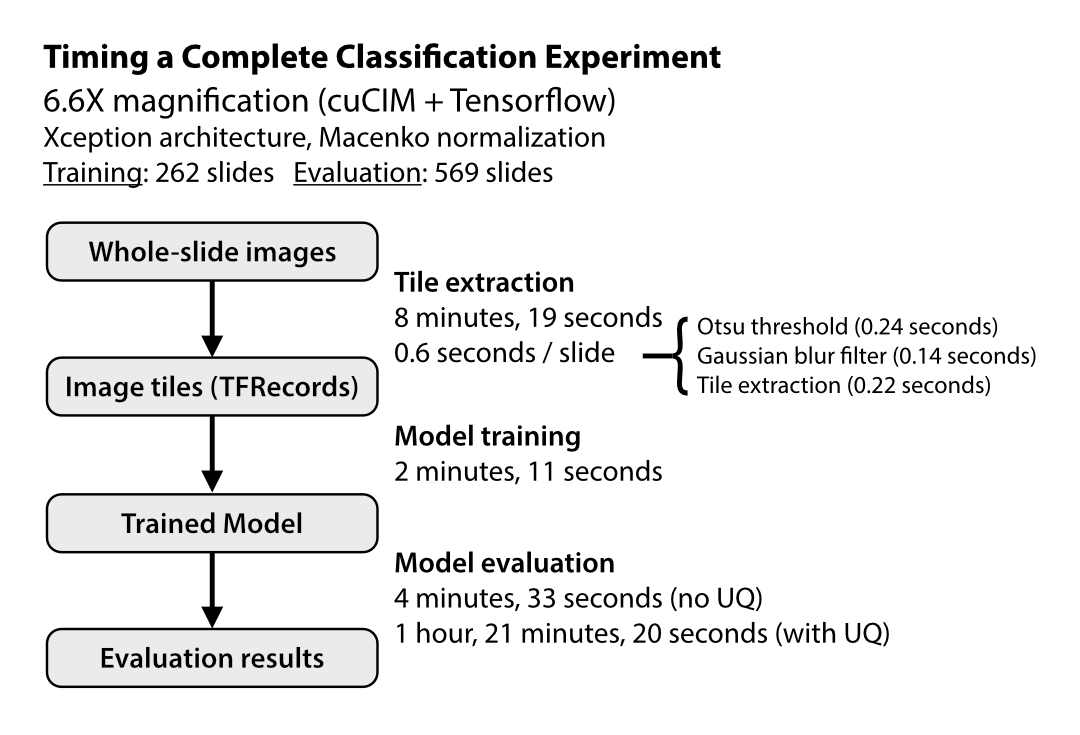
**

**Supplementary Figure 11. Runtime requirements for training and evaluating a tile-based classification model.** This figure illustrates the time required to train and evaluate a classification model using the model architecture (Xception), tile magnification (6.6X magnification, 299 x 299 pixel images), and stain normalization strategy (Macenko) described in this manuscript. The time required to process whole-slide imaging data is highly dependent upon slide reading backend, tile magnification, chosen slide filtering methods, and computational hardware. Model training and evaluation time is heavily influenced by the chosen architecture, number of epochs, stain normalization method, and GPU. Uncertainty quantification adds a significant amount of experimental time, as each image tile must pass through the network 30 times to generate an uncertainty estimate.

| **Architecture Name** | **Tensorflow** | **PyTorch** |
| --- | --- | --- |
| ResNet-18 | No | Yes |
| ResNet-50 | Yes | Yes |
| ResNet-101 | Yes | No |
| ResNet-152 | Yes | No |
| AlexNet | No | Yes |
| SqueezeNet | No | Yes |
| DenseNet | Yes | Yes |
| InceptionV3 | Yes | Yes |
| GoogleNet | No | Yes |
| ShuffleNet | No | Yes |
| ResNext50 | No | Yes |
| VGG-16 | Yes | Yes |
| VGG-19 | Yes | No |
| MobileNet | Yes | No |
| MobileNetV2 | Yes | Yes |
| MobileNetV3-S | No | Yes |
| MobileNetV3-L | No | Yes |
| Wide-ResNet50 | No | Yes |
| Xception | Yes | Yes |
| NASNet-Mobile | No | Yes |
| NASNet-Large | Yes | Yes |
| EfficientNet-V2 | Yes | No |

**Supplementary Table 1. Pre-configured image classification model architectures.** The above table lists all preconfigured model architectures supported for tile-based weakly supervised training using Tensorflow and PyTorch. In addition to these listed models, PyTorch models available from the timm package can also be utilized by specifying the desired model architecture with the prefix “timm_”. Custom architectures beyond those listed here, such as vision transformers (ViT), are also supported.

| Hyperparameter / Model Parameter | Argument | Value |
| --- | --- | --- |
| Augmentation pipeline | augment | xyrjbn |
| Batch Size | batch_size | 32 |
| Dropout probability | dropout | 0.15 |
| Early stopping | early_stop | FALSE |
| Training epochs | epochs | 1 |
| Number of hidden layers | hidden_layers | 0 |
| Use ImageNet pretrained classifier | include_top | FALSE |
| L1 regularization | l1 | 0 |
| L1 regularization (dense layers) | l1_dense | 0 |
| L2 regularization | l2 | 0 |
| L2 regularization (dense layers) | l2_dense | 0.128 |
| Learning rate | learning_rate | 5e-5 |
| Learning rate decay | learning_rate_decay | 0.98 |
| Learning rate decay steps | learning_rate_decay_steps | 1024 |
| Loss function | loss | sparse_categorical_crossentropy |
| Model architecture | model | xception |
| Stain normalizer | normalizer | macenko |
| Stain normalizer target | normalizer_source | “v3” |
| Optimizer | optimizer | Adam |
| Post-convolutional pooling | pooling | avg |
| Tile size (pixels) | tile_px | 299 |
| Tile size (microns) | tile_um | 453 |
| Uncertainty quantification | uq | TRUE |
| Mini-batch balancing (training) | training_balance | category |
| Mini-batch balancing (validation) | validation_balance | None |

**Supplementary Table 2. Hyperparameters used during weakly-supervised tile-based model training.**

| Hyperparameter / Model Parameter | Argument | Value |
| --- | --- | --- |
| Epochs | max_epochs | 20 |
| Learning rate | lr | 1e-4 |
| Weight decay | reg | 1e-5 |
| Weighted sample | weighted_sample | false |
| Seed | seed | 1 |
| Bag loss | bag_loss | “ce” |
| Bag weight | bag_weight | 0.7 |
| Model architecture | model | “clam_sb” |
| Model size | model_size | “small” |
| Dropout probability | dropout | False |
| Optimizer | opt | Adam |
| K sample | B | 8 |

**Supplementary Table 3. Hyperparameters used during weakly-supervised multiple-instance learning.**
